# ViroISDC: a method for calling integration sites of hepatitis B virus based on feature encoding

**DOI:** 10.1186/s12859-024-05763-0

**Published:** 2024-05-04

**Authors:** Lei Qiao, Chang Li, Wei Lin, Xiaoqi He, Jia Mi, Yigang Tong, Jingyang Gao

**Affiliations:** 1 College of Information Science and Technology, Beijing University of Chemical Technology, Beijing, 100029 China; 2 College of Life Science and Technology, Beijing University of Chemical Technology, Beijing, 100029 China

**Keywords:** HBV integration, Detect tools, Deep learning, Integration patterns and features

## Abstract

**Background:**

Hepatitis B virus (HBV) integrates into human chromosomes and can lead to genomic instability and hepatocarcinogenesis. Current tools for HBV integration site detection lack accuracy and stability.

**Results:**

This study proposes a deep learning-based method, named ViroISDC, for detecting integration sites. ViroISDC generates corresponding grammar rules and encodes the characteristics of the language data to predict integration sites accurately. Compared with Lumpy, Pindel, Seeksv, and SurVirus, ViroISDC exhibits better overall performance and is less sensitive to sequencing depth and integration sequence length, displaying good reliability, stability, and generality. Further downstream analysis of integrated sites detected by ViroISDC reveals the integration patterns and features of HBV. It is observed that HBV integration exhibits specific chromosomal preferences and tends to integrate into cancerous tissue. Moreover, HBV integration frequency was higher in males than females, and high-frequency integration sites were more likely to be present on hepatocarcinogenesis- and anti-cancer-related genes, validating the reliability of the ViroISDC.

**Conclusions:**

ViroISDC pipeline exhibits superior precision, stability, and reliability across various datasets when compared to similar software. It is invaluable in exploring HBV infection in the human body, holding significant implications for the diagnosis, treatment, and prognosis assessment of HCC.

## Background

Liver cancer, as reported by the World Health Organization [1], accounts for approximately 905,677 new cases and 830,180 deaths globally each year, ranking sixth in global cancer incidence and third in global cancer death rates. Its mechanism is complex and may involve multiple factors, such as viral infections [2], genetic factors [3], environmental factors, lifestyle habits, and diet [4]. There are two types of liver cancer: primary liver cancer and metastatic liver cancer. Primary liver cancer refers to a malignant tumor originating from liver cells and includes common types such as hepatocellular carcinoma (HCC), intrahepatic cholangiocarcinoma (ICC), and mixed hepatocholangiocarcinoma (MHCC) [5–8]. Among primary liver cancers, HCC is the most prevalent, accounting for 75% to 85% of cases. Chronic infection with the hepatitis B virus (HBV) [9] and hepatitis C virus (HCV) [10] are the main risk factor for the development of HCC.

HBV integrates viral DNA into the host cell genome, which is an important mechanism of HBV infection, and it can affect chromosomal stability, gene expression, DNA mutations, signal transduction pathways, and epigenetics [11, 12]. Consequently, this complex interplay can contribute to the initiation and progression of liver cancer. Investigating HBV integration offers a profound understanding of the intricate interaction between HBV and the host genome. Additionally, it enables the exploration of the heterogeneity observed in liver cell cancer, thus establishing a foundation for individualized treatment strategies. Furthermore, HBV integration is not only an important mechanism for the occurrence of HCC but also a biomarker for diagnosis, treatment [13], and prognosis evaluation of HCC [14].

The determination of integration sites plays a vital role in comprehending the mechanism of HBV integration in the human chromosome and the pathogenesis of liver cancer. Compared to other types of mutations, such as deletions or single nucleotide polymorphisms, the detection tools available for viral integration mutations are deficient. Currently, only a few tools, such as SurVirus [15] and Seeksv [16], exist for the detection of these mutations. However, these tools may experience unstable performance or fail to balance accuracy and sensitivity when faced with various coverage and integration sequence lengths. Traditional insertion mutation detection tools like Lumpy [17] and Pindel [18] may not differentiate integrated and non-integrated mutations, leading to a high number of false positives. Therefore, a comprehensive integration site detection tool with excellent performance is essential to handle the intricacies of sequencing data.

With the development of deep learning in the field of bioinformatics, an increasing number of deep learning methods have been applied in the analysis of sequencing data. For example, DeepVariant, developed by the Google Brain team, converts genomic sequences into image formats and then uses CNN to train and predict these images for mutation detection [19]. By utilizing known mutation annotation data for training, the deep learning model can grasp the features and patterns of mutations from sequencing data. Consequently, it can be utilized to predict mutations in previously uncharted sequencing data. In this study, we propose a deep learning-based method for detecting integration sites. Specifically, the approach employs natural language processing (NLP) [20] to detect integration sites by treating genome sequence alignment data as a special language. To ensure the NLP model captures the general rules and semantic information of sequence alignment data, we encode the sequence alignment data and design a feature-based HBV integration site detection channel named ViroISDC. ViroISDC exhibits excellent comprehensive performance, it achieves high precision while maintaining a relatively high sensitivity for uncovering as many HBV integrations as possible, thereby enabling subsequent HBV integration analysis. Moreover, ViroISDC ensures stable detection performance in the face of diverse HBV integration patterns, genomic variations, and the complexities associated with sequencing data.

## Results

### Model training

**Fig. 1:**
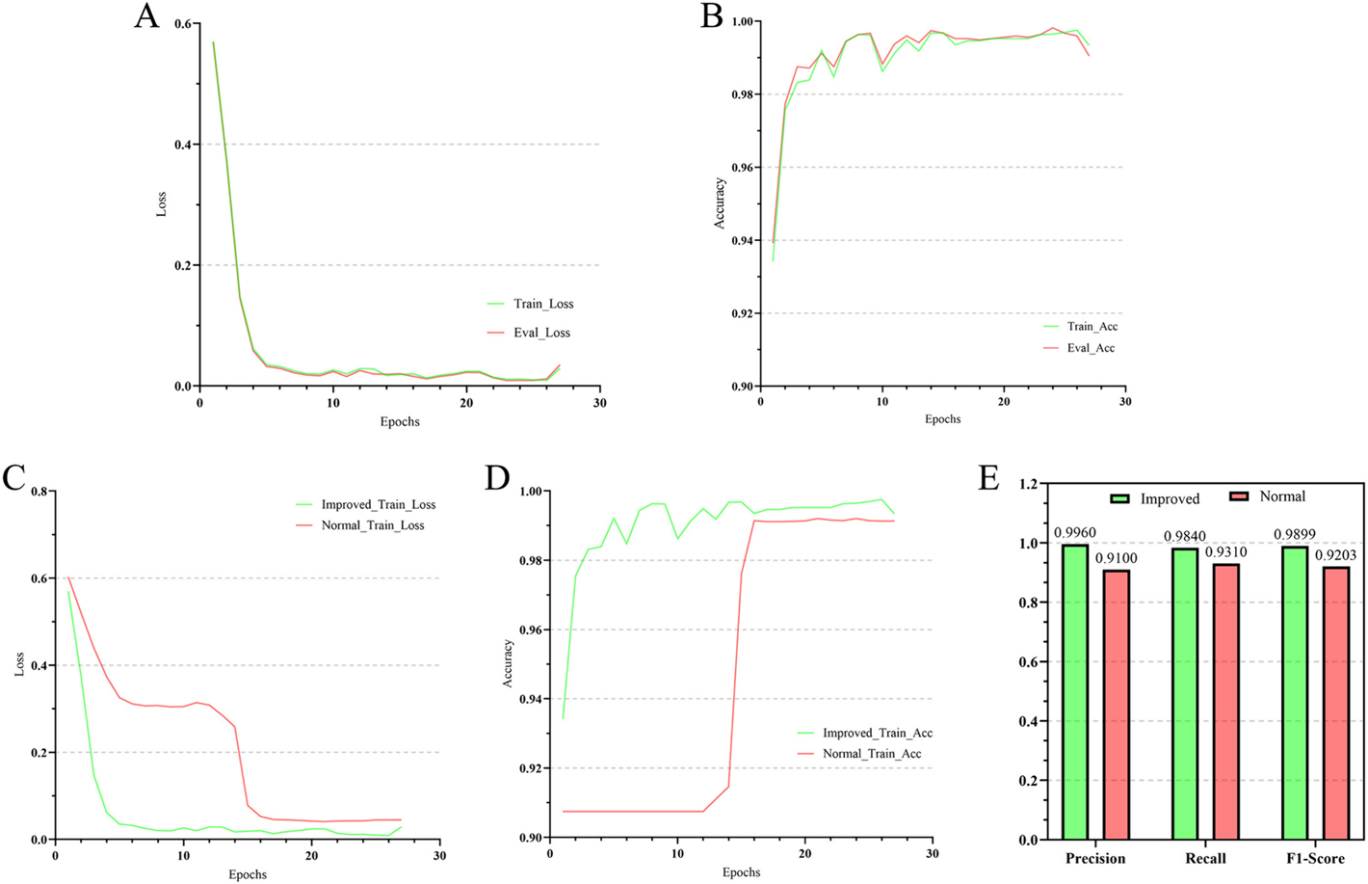
Model training and the improved model. **A** The loss curve of ViroISDC on the training set and validation set. **B** The accuracy curve of ViroISDC on the training set and validation set. **C** The loss curve of the method of extracting salient features and the ordinary methods. **D** The accuracy curve of the method of extracting salient features and the ordinary methods. **E** The comparison of the Precision, Recall and F1-score of the two models on the test set

ViroISDC was trained for 27 epochs, and the model was evaluated after each epoch, as shown in Fig. 1A, B. The model converged well by the 5th epoch, and its generalization performance was excellent, with the training and validation curves closely aligned. Moreover, considering the initial accuracy of over 85%, it can be inferred that the pretraining model successfully learned semantic information from the corpus and significantly contributed to the integration site detection task.

The method of extracting significant features is effective in improving the performance of the classification model for long alignment sequences. It is evident that the improved approach effectively accelerated the model convergence, resulting in lower loss and higher accuracy (Fig. 1C, D). Additionally, the improved model successfully mitigated the problem of gradient vanishing and exploding caused by lengthy sequences in the 15th epoch for the unimproved model, allowing the ViroISDC model to converge more smoothly. Figure 1E demonstrates the comparison of precision, recall, and F1-score on the test set for both models. The improved model outperforms the unimproved model on all three metrics. Furthermore, after analyzing the corpus data, it was found that the unimproved model struggled particularly with long sequence data, as evidenced by the majority of undetected data being longer sequences.

### Real dataset analysis

**Fig. 2:**
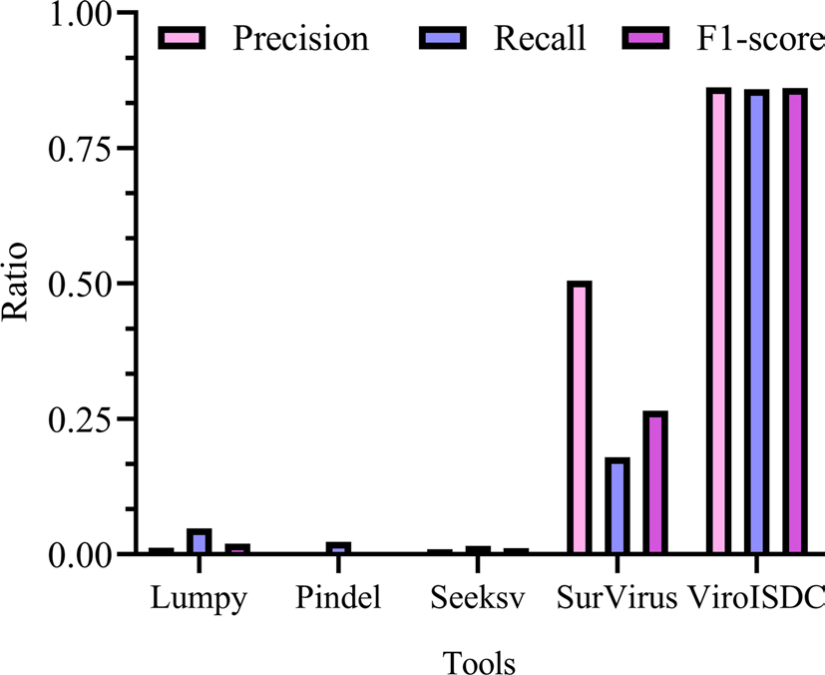
Performance of different tools on real datasets

As shown in Fig. 2, for the real dataset SRA335342, Lumpy, Pindel, and Seeksv detected only a relatively small number of integration sites, resulting in poor performance in terms of Precision, Recall, and F1-score. Among the five compared tools, SurVirus ranked second in performance, with a Precision of 50.54%, Recall of 17.94%, and an F1-score of 26.48% by combining Precision and Recall. Based on these three indicators and SurVirus' detection results, it can be concluded that SurVirus achieved relatively high accuracy in detecting integration sites. However, its sensitivity was relatively low, indicating that it is more conservative and could only detect a small number of integration sites. In contrast, ViroISDC performed better than other tools in all three indicators in this real dataset, with a Precision of 86.21%, Recall of 85.88%, and an F1-score of 86.04%. The detection results demonstrated that ViroISDC accurately and reliably identified integration sites while also exhibiting higher sensitivity in detecting more integration sites. This characteristic is particularly valuable in uncovering important features related to hepatitis B virus integration during downstream analysis of integration sites.

### Comparison with different detection methods in simulated dataset

To better illustrate the good performance of our proposed method, we introduce currently widely used detection methods, including Lumpy, Pindel, Seeksv, and SurVirus. Lumpy is a probability-based framework that uses breakpoint evidence to determine the location and type of structural variation by combining different structural variation signals. The Pindel algorithm uses a pattern growing method to find breakpoints in the genome sequence and is used to detect large deletions and medium-sized insertions. The Seeksv algorithm is based on four different detection signals: soft-clipped reads signal, discordant paired-end mappings signal, document (DOC) signal and fragments that cannot be matched at both ends. signals, Seeksv comprehensively utilizes these signals to detect structural variations and viral integration breakpoints. SurVirus uses a repeat-aware alignment algorithm that more accurately aligns reads to host and viral genomes. This algorithm can correct erroneous alignments in repeated regions, thereby improving the accuracy of integration detection.

**Fig. 3:**
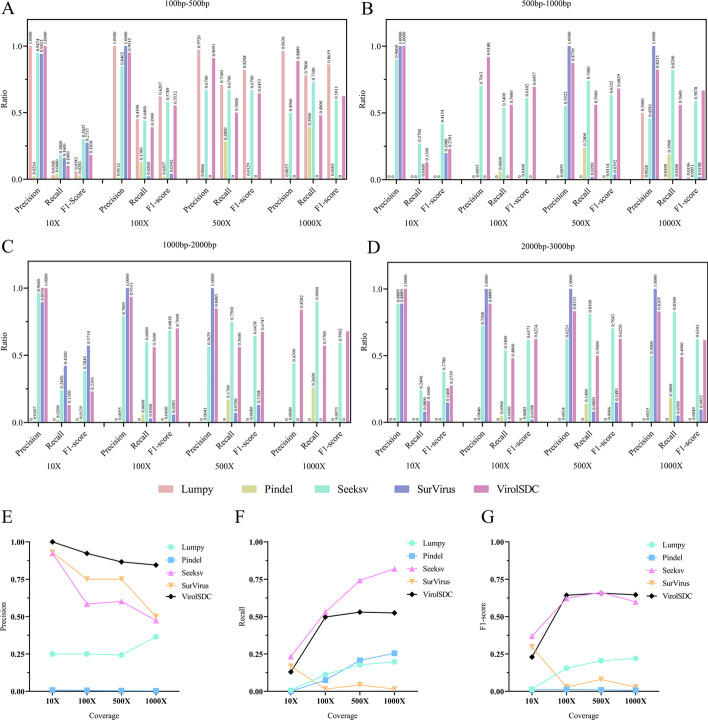
Performance of different tools for simulated different sequencing depths and insertion sequence lengths. **A**–**D**, the performance of different tools for different lengths of insertion sequences at sequencing depths of 10X,100X, 500X and 1000X, respectively. **E**–**G** performance of different tools in detecting integration sites under different sequencing depth

Given the uncertainty of integration events in the real dataset due to the nature of sample sequencing and potential errors during the process, as well as the lack of a gold standard, simulation datasets were employed in this study to evaluate and analyze the experiment. The integration site detection performance of five tools, namely Lumpy, Pindel, Seeksv, SurVirus, and ViroISDC, was assessed with simulated datasets (Fig. 3).

In terms of Lumpy's integration length between 100 bp-500 bp, the precision of the four coverage levels is relatively high. Among them, 10X and 100X have a precision of 1, while 500X and 1000X have a precision of 97.26% and 96.3%, respectively. However, Lumpy is almost ineffective in other integration lengths, with only 50% accuracy in integration between 500 bp-1000 bp at a coverage level of 1000X. Pindel shows the ability to detect most coverage levels and integration lengths but with a relatively poor detection performance. Conversely, Seeksv, SurVirus, and ViroISDC show better detection performance overall. Seeksv performs well in low coverage levels and short integration situations. SurVirus has a relatively high overall precision rate, although its sensitivity is often lower. Moreover, SurVirus is ineffective in detecting integration between 500 bp-1000 bp at a coverage level of 100X, 100-500 bp at a coverage level of 500X and 1000X, and 1000-2000 bp at a coverage level of 1000X. ViroISDC demonstrates good performance in different integration lengths under full coverage levels. It maintains high levels of accuracy and sensitivity, making it advantageous for most coverage levels and integration lengths.

Additionally, we investigated the changes in detection performance of integration sites across different tools under varying coverage. ViroISDC consistently outperforms other tools in accuracy, followed by SurVirus. Moreover, ViroISDC exhibits a relatively small fluctuation amplitude while retaining high accuracy, indicating that ViroISDC is less influenced by coverage (Fig. 3E). The recall rate curve indicates that all tools, except SurVirus, showed an increase in recall rate as coverage increases. Out of all the tools, Seeksv performs the best, closely followed by ViroISDC (Fig. 3F). And from the perspective of F1-score indicator, ViroISDC outperforms other tools in most cases (Fig. 3G).

### Sequence verification near the integration site

**Fig. 4:**
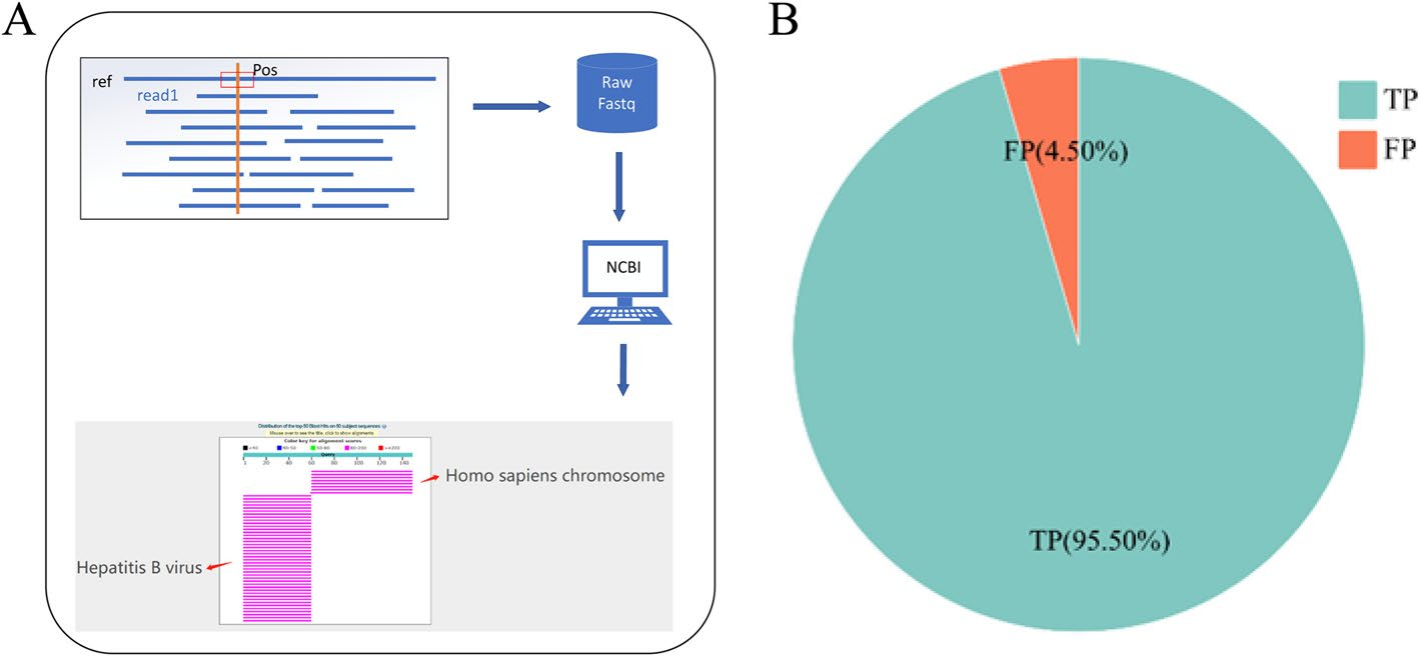
Verification of integration sites. **A** Verification workflow of the the sequence near integration site. **B** Accuracy of predicted integration sites. TP represents true positive (integrated sites are true), and FP represents false positive (integrated sites are false)

To further verify the effect of ViroISDC in detecting HBV integration, we randomly selected 200 integration sites for manual verification. The verification process is shown in Fig. 4A, which is a description of the sequence verification near a certain integration site. After obtaining the integration site, extract 10 bp before and after the integration site, and extract reads containing the above related sequences from the sequencing data. In NCBI, these reads are compared with the Nt database [21] for BLATSTn [22]. This tool will display the alignment results in the form of a picture, and it can also detect the alignment information of each sequence, including identity, similarity, sequence length, etc. Based on this information, we verified the sequences near the integration site. The results of this figure show that these two sequences are compared with the hepatitis B genome and the human genome. And the location of the breakpoint is the same as what we found, indicating that the location of the breakpoint and the sequence before and after are accurate. We also collected the verification results of these 200 integration sites, with positives (TP) accounting for 95.5% and false positives (FP) accounting for 4.5% (Fig. 4B). The results show that ViroISDC has good detection performance and reaches the expected accuracy level.

### Correlation between HBV integration and chromosome

**Fig. 5:**
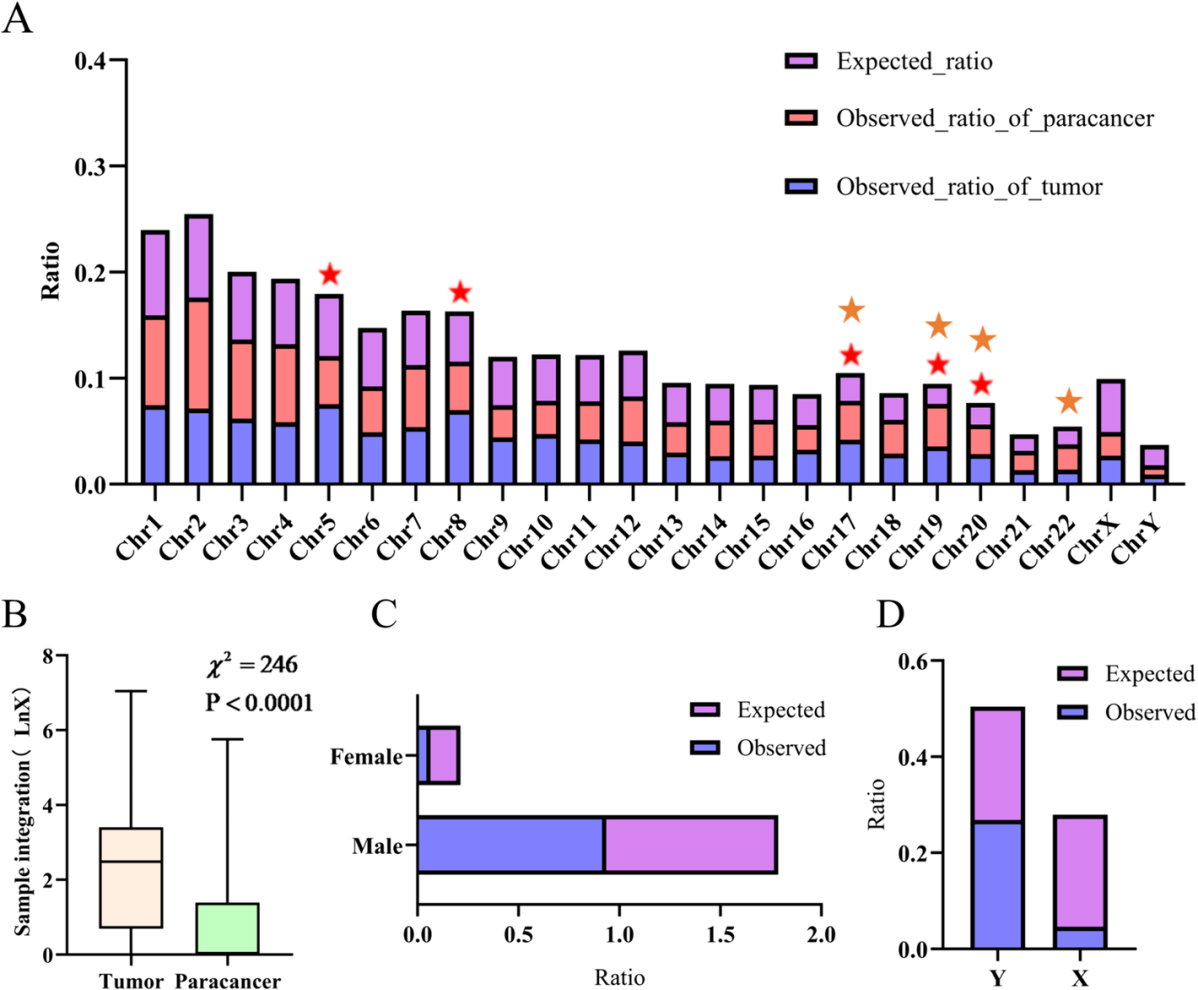
Distribution of HBV integration sites. **A** Distribution of HBV integration sites on human chromosomes. **B** Distribution of integration sites on cancer tissue and paracancerous tissue. **C** Distribution of HBV integration sites in different gender. **D** Distribution of HBV integration on sex chromosomes

Integration site detection was performed on 289 HCC patients, resulting in the identification of 6238 integrated sites in cancer tissues and 1250 integrated sites in paracancer tissues. In liver cancer tissues, integrated sites were distributed in 24 chromosomes. The count of integrated sites on each chromosome was normalized. Compared to the uniformly distributed normalized data, HBV integration on chromosomes showed a certain selection bias (Fig. 5A). In cancer tissues, the preferentially integrated chromosomes were marked with red pentagrams, including chromosome 5 (7.53% VS 5.84%, ${\upchi }^{2}=32.781>3.84$, $P=1.03\times {10}^{-8}<0.05$), chromosome 8 (6.99% VS 4.73%, ${\upchi }^{2}=71.977>3.84$, $P=2.18\times {10}^{-17}<0.05$), chromosome 17 (4.18% VS 2.62%, ${\upchi }^{2}=60.501>3.84$, $P=7.35\times {10}^{-15}<0.05$), chromosome 19 (3.56% VS 1.91%, ${\upchi }^{2}=90.885>3.84$, $P=1.52\times {10}^{-21}<0.05$), and chromosome 20 (2.82% VS 2.04%, ${\upchi }^{2}=19.298>3.84$, $P=1.12\times {10}^{-5}<0.05$). The comparison between actual experimental HBV integration in paracancer tissues and theoretical HBV integration unveiled a certain chromosomal selection bias in HBV integration. The preferentially integrated chromosomes were marked with orange pentagrams in Fig. 5A, including chromosome 17 (3.68% VS 2.62%, ${\upchi }^{2}=6.286>3.84$, $P=0.0121<0.05$), chromosome 19 (4% VS 1.91%, ${\upchi }^{2}=32.29>3.84$, $P=1.33\times {10}^{-8}<0.05$), chromosome 20 (2.8% VS 2.04%, ${\upchi }^{2}=4.082>3.84$, $P=0.0434<0.05$), and chromosome 22 (2.4% VS 1.66%, ${\upchi }^{2}=5.081>3.84$, $P=0.0242<0.05$).

Combining analysis of cancer tissue and paracancer tissue, it was evident that chromosomes 7, 19, and 20 were all susceptible to invasion by the hepatitis B virus. In order to investigate whether there is an infection advantage of hepatitis B in the two types of tissue, the distribution of HBV integration sites in the two types of samples was statistically analyzed in the experiment, as shown in Fig. 5B. Notably, HBV integration in cancerous tissue and paracancerous tissue exhibited significant differences (χ^2^ = 32.29 > 3.84, *P* < 0.0001), with a greater propensity for the hepatitis B virus to integrate into cancerous tissue. This observation may be attributed to specific genes or genomic regions on the chromosomes, such as the inactivation of tumor suppressor genes which leads to the proliferation of cancer cells, and the integration sequence embedded in cancer cells will rapidly proliferate together with cancer cells, resulting in more integrations being detected. Therefore, the analysis of HBV integration of samples can locate the foci of liver cancer and determine the degree of liver cancer development, which is important for the prevention, diagnosis and treatment of liver cancer.

### Correlation between HBV integration and gender

In 289 HCC patients, there were a total of 43 females and 246 males. Among the female samples, 485 integrated loci were detected, while 7003 were detected in the male samples. Compared to the expected distribution (no gender bias in HBV integration), HBV integration exhibited significant gender bias (${\upchi }^{2}=417.225>3.84$, $P=9.81\times {10}^{-93}<0.05$), indicating that the hepatitis B virus is more likely to invade the male body (Fig. 5C). It is consistent with previous observations and proves the feasibility and accuracy of our method. To further study the correlation between integration and gender, the experiment analyzed the distribution of integrated loci on the sex chromosome number 24, as shown in Fig. 5D. A total of 66 integrated sites were found on the chromosome Y and 2 on the chromosome X. When combined and normalized, the distribution of integrated loci on the sex chromosomes was obtained. Comparing the expected distribution with the actual distribution, it was found that the hepatitis B virus is more likely to integrate into the chromosome Y(${\upchi }^{2}=7.503>3.84$, $P=0.006<0.05$), which corresponds to the conclusion that the hepatitis B virus is more likely to invade the male body.

### Distribution of HBV integration on genes and chromosomes

**Fig. 6:**
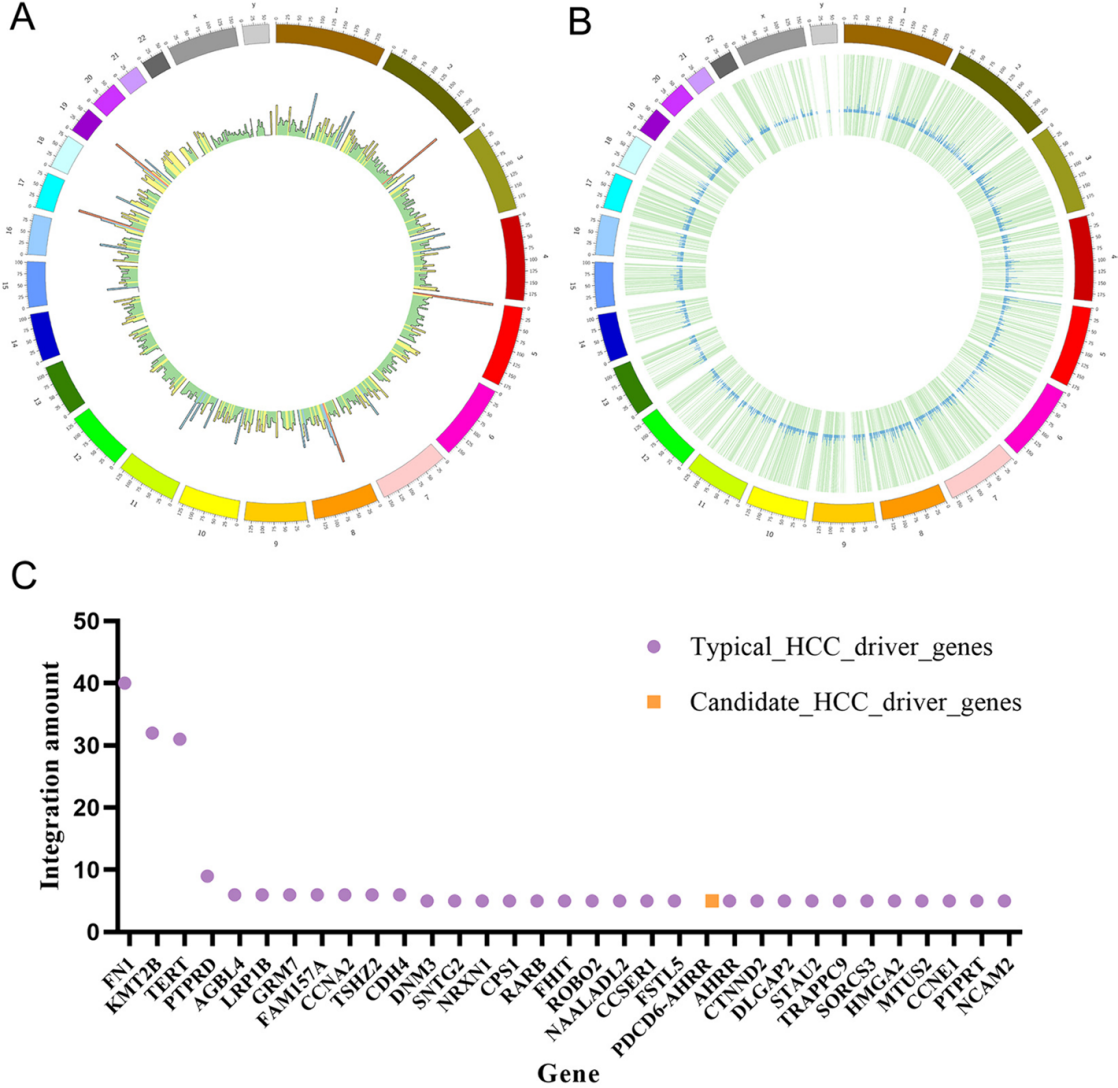
Analysis of HBV high-frequency integration genes. **A** Distribution of HBV integration sites on human chromosomes. The integration amount of each scale was represented by a small bar graph, with colors divided into four gradients: red (high integration), blue (relatively high integration), yellow (relatively low integration) and green (low integration). **B** Distribution of HBV integration sites on genes. The chromosome regions where genes with integration sites were located and represented with light green bars, with the integration amount of genes represented by small blue bar graphs. **C** Integration frequency of high-frequency integrated genes

To further explore the mechanism of HBV integration-induced carcinogenesis, we investigated the distribution of integration sites. It was observed that HBV had a high integration frequency in the 220 M-225 M interval of chromosome 2, the 0 M-5 M interval of chromosome 5, the 45 M-50 M interval of chromosome 8, the 20 M-25 M interval of chromosome 17, and the 35 M-40 M interval of chromosome 19 (Fig. 6A, B). Notably, these intervals mainly involved regions associated with liver cancer, such as cancer driver genes, tumor suppressor genes or telomeres [11].

Through setting a threshold (with integrated site number greater than or equal to 5), 33 high-frequency integrated genes were selected, as shown in Fig. 6C. Further comparison of these 33 high-frequency integrated genes with liver cancer-related NCG [23] and HCCDB [24] databases revealed that 32 of them were present in these databases. Among these 33 high-frequency integrated genes, the integrated site number of FN1, KMT2B, and TERT was much higher than that of other genes. FN1 is located in the region of 210 M-230 M on chromosome 2, and it is related to the expression of a protein that affects cell differentiation, proliferation, wound repair, and cell adhesion, among other processes. The abnormal expression of FN1 has been proven to be associated with liver cancer [25]. Considering the high integration of HBV in the FN1 region, it can be inferred that the integration of the hepatitis B virus into the FN1 gene may influence protein expression, thereby promoting the development of liver cancer. KMT2B is located in the interval of 40 M-50 M on chromosome 19, and it is a gene that affects DNA repair and cell cycle, and at the same time is an important driving gene for liver cancer. TERT is located in the region of 0 M-5 M on chromosome 5, and it encodes telomerase reverse transcriptase, which can maintain chromosome stability and affect cell differentiation and proliferation. The impact of TERT on liver cancer is speculated to be due to the integration of hepatitis B virus affecting the stability of chromosomes.

In addition, a high-frequency integration gene called PDCD6-AHRR was detected in the experiment, which is not included in the NCG and HCCDB databases. This gene, after excluding the 32 high-frequency integration genes found in the databases, may also play a role in the occurrence and development of HBV integration and liver cancer.. PDCD6-AHRR is a special gene formed by the rearrangement of two genes, PDCD6 and AHRR, and is a fusion gene. Among them, PDCD6 mainly affects various biological processes such as cell apoptosis, division, and endoplasmic reticulum stability, while AHRR mainly affects immune response and other biological mechanisms. The recombination of these two types of genes into PDCD6-AHRR may affect the occurrence and development of liver cancer, making it a valuable target for liver cancer research.

Through the analysis of high-frequency integration genes, it was found that HBV integration predominantly promotes the transformation of cells into liver cancer cells by affecting the expression of liver cancer driver genes, inactivating tumor suppressor genes, and changing the structure and stability of the genome. Subsequent proliferation of liver cells with integration sequences ultimately leads to the occurrence and progression of liver cancer.

## Discussion

As HBV integrations may be specific biomarkers for prediction of clinical outcomes, However, rapid, reliable and stable HBV integration site detection processes are rare [26]. In this study, a new HBV integration site detection tool based on feature coding called ViroISDC is presented, which is a natural language processing method. By establishing data generation rules for the corpus, genetic sequencing data is transformed into a corpus-like natural language format, and feature coding is applied to the corpus. The encoder in the Transformer is used to extract semantic information and general rules from genetic sequencing data, and then perform integration site detection. ViroISDC demonstrates superior performance compared to other tools in terms of precision, recall, and F1-score for both real and simulated datasets, exhibiting remarkable stability and reliability. It is minimally affected by variations in sequencing depth and integration length. Besides, ViroISDC plays a significant role in understanding the interaction between HBV and the host genome, exploring the pathogenesis of liver cancer, and investigating the heterogeneity of liver cell carcinoma. Furthermore, the integration sites identified through ViroISDC's genome sequencing data offer enhanced support and assistance for the diagnosis, personalized treatment, and prognostic evaluation of HCC.

Previously, studies on the integration preference of HBV on different chromosomes have shown that HBV integration into chromosomes 2 and 17 is more likely to result in male tumors [7]. In this study, a comprehensive analysis revealed that in cancerous tissues, HBV tends to integrate into chromosomes 5, 8, 17, 19, and 20, while in paracancer tissues, HBV prefers integration into chromosomes 17, 19, 20, and 22. Moreover, cancerous tissues exhibit a higher integration frequency compared to paracancer tissues. Consistent with previous findings [27], ViroISDC successfully detected HBV integration hotspots such as TERT and KMT2B, indicating the software's accuracy in predicting integration sites. Additionally, besides the commonly reported HBV integration genes, this study discovered a significant number of HBV integration sites on the PDCD6-AHRR gene. Although not included in the NCG and HCCDB databases, this gene holds considerable value for understanding the occurrence and development of liver cancer. The distribution and frequency of HBV integration sites in human chromosomes and genes suggest that HBV may integrate into regions related to liver cancer driver genes, tumor suppressor genes, and other pertinent areas. By influencing the expression of these liver cancer-related genes and the stability of the genome, HBV can induce the transformation of normal cells into cancer cells. The subsequent proliferation and development of these transformed cells contribute to the progression of hepatocellular carcinoma.

Besides, we also found that ViroISDC could be improved. First, the specific database used in this study to retrieve relevant reference sequences and gene information may not be completely comprehensive or accurate, which may result in some HBV integration sites not being detected. Then, this method uses an encoder in the Transformer architecture to extract semantic information and general rules. However, the extraction of semantic information may be affected by corpus data quality and feature encoding, which may introduce errors and uncertainties.

Regarding the future work, we are dedicated to designing a robust graph neural network model to investigate whether genes at HBV integration sites could serve as potential therapeutic targets for liver cancer. Notably, the research outcomes from the iGRLDTI [28] and FCAN-MOPSO [29] studies have provided promising insights into exploring HBV integration site genes as therapeutic targets for liver cancer. The former proposes an improved graph-based learning method that successfully solves the problems of over-smoothing and non-aggregation of information in the prediction process. The latter highlighted the advantages of a graph clustering algorithm, which shows better accuracy and convergence on real complex networks of different sizes. Furthermore, GKLOMLI [30] pointed out that using network similarities to build neural networks can help comprehensively consider more entities and contextual information. Building upon these research advances, we aim to continue our in-depth exploration with the goal of developing a more precise and efficient graph neural network model, offering innovative strategies and methodologies for the treatment of liver cancer.

## Conclusions

ViroISDC is a novel feature encoding-based tool for detecting HBV integration sites. In comparison with similar software, ViroISDC exhibits superior precision, stability, and reliability across various datasets. When employed for downstream analysis, ViroISDC proves invaluable in exploring HBV infection in the human body, holding significant implications for the diagnosis, treatment, and prognosis assessment of HCC.

## Methods

### Workflow of ViroISDC

**Fig. 7:**
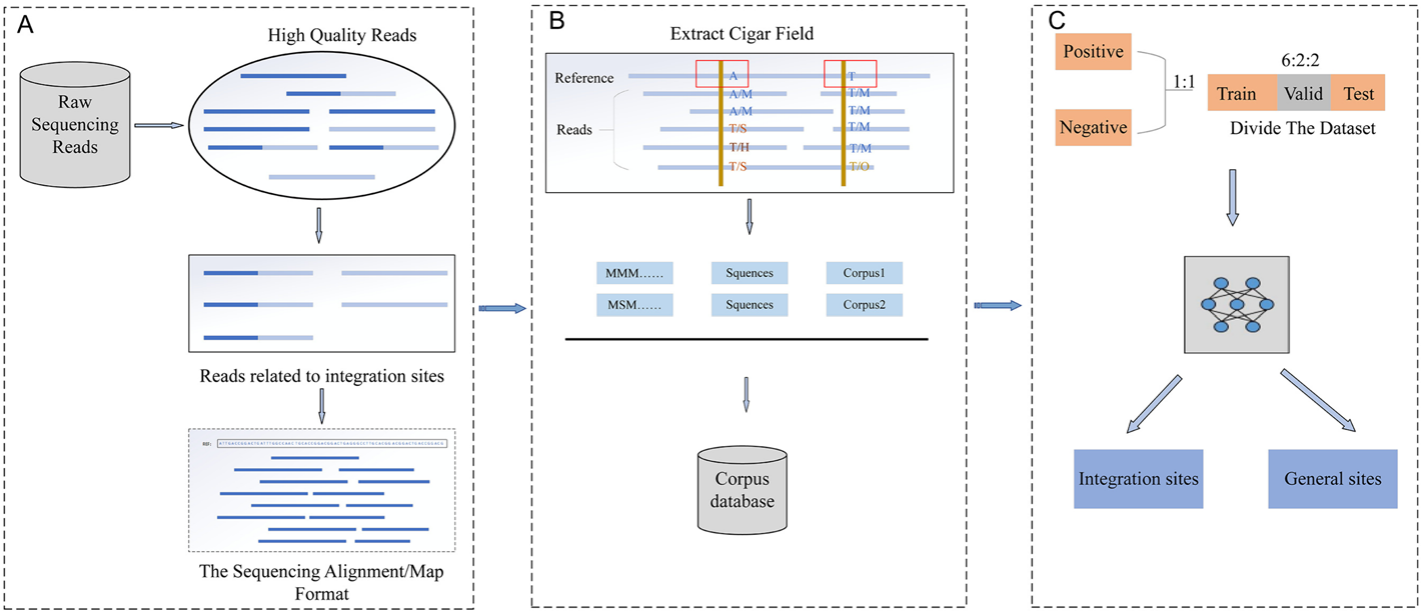
Overview of ViroISDC workflow. **A** Data preprocessing. **B** Corpus data generation. **C** Integration site prediction

ViroISDC is an HBV integration site detection pipeline based on feature encoding. Its core detection principle involves encoding the features in the CIGAR field and sending the resultant encoded word vector to an NLP model for integration site detection. The workflow of ViroISDC, as depicted in Fig. 7, is divided into three parts: data preprocessing, corpus data generation, and integration site prediction. The third part has two distinct phases, the training phase and the final application phase. The training phase performs training and prediction based on the divided dataset. The application phase, on the other hand, directly uses the trained model to predict samples and generate result sets for each sample.

### Sequencing data processing and corpus data generation

ViroISDC's integration site detection model requires specific language data as input. The raw sequencing data cannot be used as input for the model as they are simply unordered reads. Therefore, it is necessary to transform the raw sequencing data into corpus data. The processing flow of the original sequence was shown in Fig. 7A. This process was designed according to the method of Li et al. [31]. In brief, quality control of raw data was performed with Trimmomatic. The high-quality reads were aligned with the 10,791 reference sequences of HBV (NCBI Genbank, 2022) to obtain reads related to the integration of HBV. The HBV-related reads were then aligned with the human reference sequence (GRCh37) to obtain the Human-HBV related reads.

To generate corpus data, a window of size 3 is opened, utilizing human reference sequences as coordinates. Within this window, the CIGAR field is extracted, and its contents are concatenated to construct a corpus. The sliding step of the window is set to 1.

### Corpus data feature coding

Although the corpora extracted in the previous text are sequences, they can be seen as a special language composed of sequence alignment features. Encoding this special language and inputting it into an NLP model can unveil the relationships between sequence information and their expressions. However, a current challenge lies in the inability of computers to directly process human language or feature sequences and comprehend their semantics and meaning. In order to solve this problem, a way of encoding the feature sequence is proposed (Fig. 7B). Text data in NLP tasks exists discretely in the form of words or characters, so these words or characters can be encoded into vector form which is computable by computers. The commonly used encoding methods include k-mer encoding, one-hot encoding and word embedding [32].

The k-mer encoding is to divide the DNA sequence into subsequences of length k, and map each subsequence to a number or vector. However, due to the large size of virus and human genetic data, the dictionary will be too large and the computing and storage overhead will increase. One-hot encoding represents each base (A, T, C, G) with an orthogonal vector, in which only one element is 1 and the other elements are 0. This means that in this encoding method, different amino acids or genes are completely independent and have no correlation with each other because they are encoded into different, orthogonal vectors. Therefore, it is precisely because of the nature of one-hot encoding that it is impossible to express the correlation between amino acids or genes. If we hope to capture correlations between amino acids or genes, we need to use better coding methods.

**Fig. 8:**
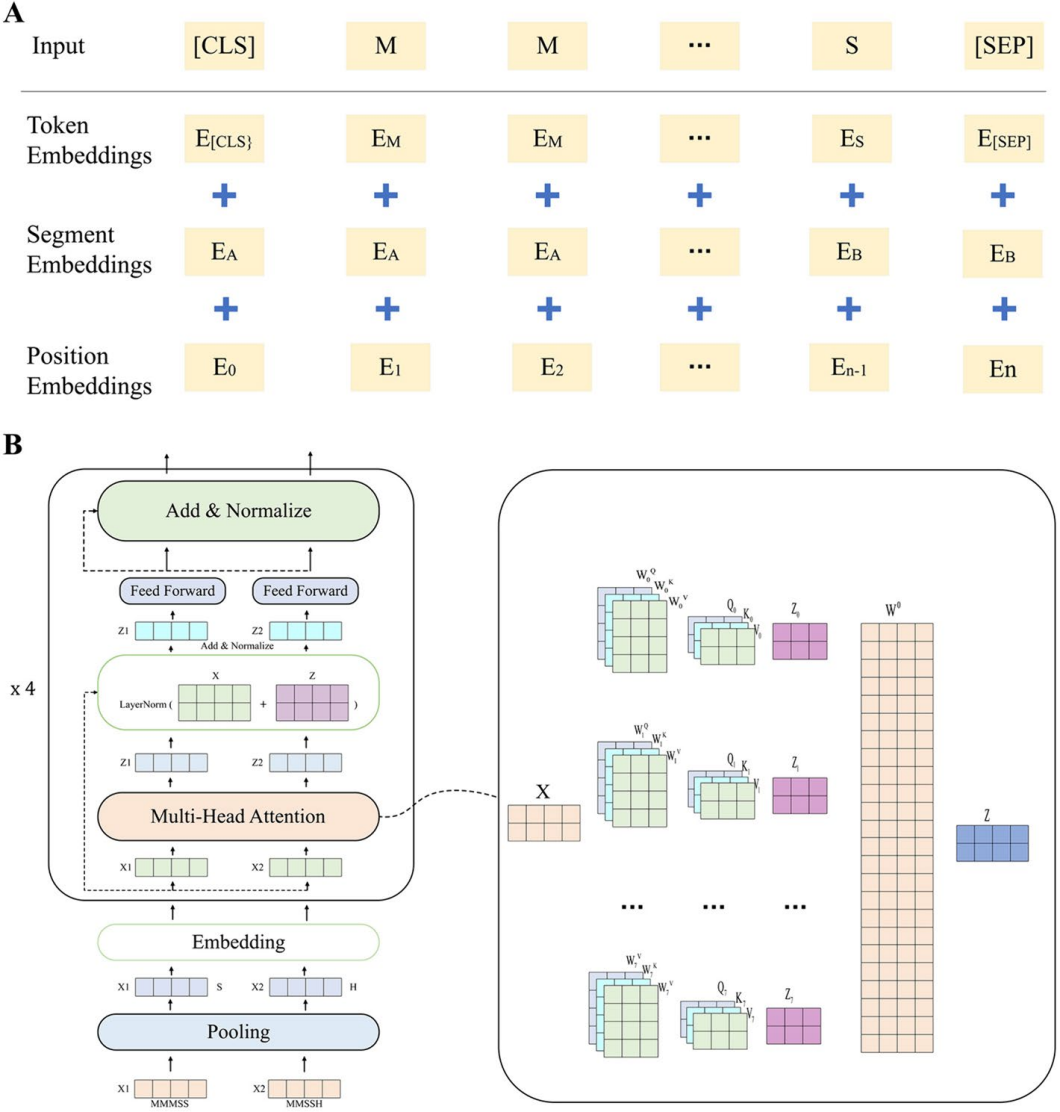
Details of corpus data feature coding and ViroISDC structure diagram. **A** Composition of word embedding. **B** Overall model structure diagram of ViroISDC and details of the long head attention

The encoding method we proposed in this article not only improves the semantic effect, but also effectively shortens the length of the sentence. ViroISDC considers the semantic information among different comparison features and utilizes Bert's pre-trained model to convert the vocabulary of each feature sequence into a word vector. This process is known as word embedding, which enables the mapping of discrete textual words to a low-dimensional space vector representation, as shown in Fig. 8A. The resulting low-dimensional vector not only captures the meaning of the word itself but also encompasses the positional relationship between words and the relationships among words within the sentence. The word vectors in this paper comprise three components: Token Embedding, Segment Embedding, and Position Embedding. Token Embedding represents the vector representation of the words in the feature vocabulary, learned during the model's pre-training process. On the other hand, Segment Embedding serves as sentence embedding, enabling the distinction between the encoding of different sentences. Lastly, Position Embedding provides positional embedding, complementing the temporal information of the text.

CIGAR is a value that describes the comparison state, which has 9 states. Among them, the states related to HBV integration are mainly M (complete match), S (soft clipping), and H (hard clipping). Other states can be considered as noise for HBV integration site detection. In order to make ViroISDC more accurate in detecting integration sites, noise is uniformly set as O (representing others) in ViroISDC. Additionally, the sequencing depth fluctuates greatly due to factors such as different sample sizes during sample collection, varying lengths of sequences, and different numbers of PCR amplifications. The length of the linguistic data also varies, ranging from approximately 30 to 3000 words. However, NLP generally performs poorly on lengthy sentences, such as Bert, which yields better results when processing sentences consisting of fewer than 512 words. Consequently, ViroISDC employs a method that involves extracting significant features for sentence encoding. Specifically, it involves two steps: word formation and extracting salient features.

Firstly, word formation combines every five words to form a new word. For instance, "MMMSS HHSMM" is two newly-formed words. This method reduces the original sentence by five times immediately. However, word formation can lead to a new problem—an explosion in vocabulary. The use of M, S, H, and O to form words theoretically produces ${4}^{5}$ different types of new words. Many of these newly created words rarely appear in a sentence, and adding them to the vocabulary will only complicate the problem. Based on the characteristic of using PCR amplification of data during sequencing, rules were defined to extract significant features after word formation, as shown in Table 1. If a word is composed of multiple different features, the effects of each feature vary. Judgments are made in the order of H > S > O > M. Specifically, if a word contains the H feature, it is set to H. If a word does not contain the H feature but has the S feature, it is set to S. Mixed-type words are handled similarly. If a word is composed of the same feature, it becomes a word of the same type, and the word is set to the value of that feature.

**Table 1:** Word formation rules

Word type	Rules
Hybrid	H > S > O > M
Homotype	Set to the characteristics that make up the word, such as H, S, M, O

The rules set above are actually equivalent to a kind of limiting maxpooling operation, which can effectively reduce the size of the vocabulary and also has the function of eliminating noise and highlighting variable features.

### Integrated site detection model construction

ViroISDC is an NLP-based HBV integration site detection pipeline, with its core being an NLP model that incorporates the encoder part of the Transformer [33], as depicted in Fig. 8B. The model takes a series of corpora as input, which undergoes custom rule-based pooling and embedding layers before being fed into four encoder layers. Finally, a fully connected layer is applied for classification on the hidden layer of the last encoder. Within the encoder, there are three main stacked structures: multi-head self-attention layer, normalization layer, and feed-forward layer.

The multi-head self-attention layer is the pivotal component of the entire model, capable of capturing the interdependencies between different spatial positions in a sentence. ViroISDC employs 8 attention heads, which effectively create 8 learning spaces, each capable of attending to distinct key points.

In the Fig. 8B, X represents the word vector matrix in the input network, with each row representing the embedding of a word vector. The input vector X is used to compute the query $Q_{i}$, key $K_{i}$, and value $V_{i}$ (where i denotes the i-th attention head) through Eq. (1).

1 $$Q_{i} = XW_{i}^{Q} ,\;K_{i} = XW_{i}^{K} ,\;V_{i} = XW_{i}^{v}$$

The weight matrices $W_{i}^{Q} ,{ }W_{i}^{K}$ and $W_{i}^{V}$ correspond to the three linear transformation operations. By plugging in the values of $Q_{i}$, $K_{i}$, and $V_{i}$ into Eq. (2), we can calculate the output $Z_{i}$ for the i-th attention head in the attention space.

2 $$Z_{i} = Attention(Q_{i} ,\;K_{i} ,\;V_{i}) = softmax(\frac{{Q_{i} K_{i}^{T} }}{{\sqrt {d_{k} } }})V_{i}$$

The correlation between vectors in matrices $Q_{i}$ and $K_{i}^{T}$ is computed through inner product, representing the interrelation among the vectors (and the interrelation among individual words). After applying softmax normalization, the resulting values are multiplied by matrix $V_{i}$ to obtain the expression matrix $Z_{i}$ for each dimension. The outputs from the 8 attention heads are concatenated and multiplied by the weight matrix $W^{0}$ of a linear layer to yield the output Z of the multi-head self-attention layer, as depicted in Eq. (3).

3 $${\text{Z}} = Concat (Z_{0} ,\;Z_{1} ,\; Z_{2} , \ldots , Z_{7} )W^{0}$$

### Model training

The model training process consists of two main stages: the first stage involves pretraining the bioinformatics language model, and the second stage involves fine-tuning the model for the integration site detection task.

During the pretraining stage, ViroISDC utilizes a large-scale of unlabeled base-pair alignment information for unsupervised pretraining, creating a specialized pretraining model for bioinformatics. The pretraining process primarily includes random feature masking and next-site prediction, enabling the pretraining model to understand the specific language of sequence alignment information. The HBV integration site detection model is then fine-tuned based on this pretraining model. The integration site detection task is a dichotomous problem, distinguishing integration sites from regular sites. The training data for the model consists of 6,834 manually annotated integration site samples. Initially, these samples are used as positive instances, and then random regular site samples are extracted from the upstream or downstream regions of these integration sites to serve as negative instances. Finally, positive and negative instances are mixed in a 1:1 ratio to create the benchmark dataset. During the fine-tuning stage of the ViroISDC model training, the dataset is split in a 6:2:2 ratio, as illustrated in Fig. 7C.

### Evaluation metrics

In this experiment, the detection performance of ViroISDC in integration sites was evaluated using three metrics: precision, recall, and F1-score. Precision, defined as TP/(TP + FP), represents the ratio of true positive integration sites predicted by the tool compared to the total predicted integration sites. Recall, defined as TP/(TP + FN), represents the ratio of true positive integration sites predicted by the tool compared to the actual integration sites. F1-score, a harmonic mean metric, combines information from both precision and recall to evaluate the overall performance of the tool in integrating the two metrics. It is calculated as 2*Precision*Recall/(Precision + Recall). Based on these 3 metrics, we compared ViroIDSC with four commonly used tools: Lumpy, Pindel, Seeksv and SurVirus.

### Dataset

Four datasets, consisting of three real datasets and one simulated dataset, are utilized in this study to comprehensively evaluate the performance of ViroISDC, as shown in Table 2. The first dataset was obtained through Illumina sequencing and was originally published by the Beijing Institute of Genomics (NCBI BioProject accession: PRJNA298941). This dataset contains sequencing data from cancer tissue and paracancer tissue of 426 HCC patients. The second and third datasets, Cap and Plasma, were generated in collaboration with the biological laboratory at Beijing University of Chemical Technology. These datasets include sequencing data from cancer and paracancerous tissues, as well as six sets of plasma sequencing data.The sequencing data of the second and third datasets were under the Bioproject ID PRJNA1011497 and they can be retrieved in the SRA (Sequence Read Archive, https://www.ncbi.nlm.nih.gov/sra) with run SRR26172074, SRR26172075, SRR26172076, SRR26172077, SRR26172078, SRR26172079, SRR26172080, SRR26172081, SRR26172082, and SRR26172083, respectively. These datasets include sequencing data from cancer tissue and paracancer tissue, as well as six sets of plasma sequencing data, as shown in Table 2. Finally, the simulated dataset was designed using wgsim (https://github.com/lh3/wgsim). The simulated dataset comprises 16 sample data based on 10,791 hepatitis B reference sequences. Four different depths of data were simulated, including 10x, 100x, 500x, and 1000x, with four integrated sequences of varying lengths (100 bp-500 bp, 500 bp-1000 bp, 1000 bp-2000 bp, 2000 bp-3000 bp) for each depth, and each sample simulating 100 integrated events. Using these datasets, a meticulous and accurate evaluation of ViroISDC's performance can be conducted.

**Table 2:** Plasma and cap datasets

Dataset name	Type	Source	Strategy	Selection	Layout
SRR26172074	Plasma6	Genomic	Targeted-capture	Random	Paired
SRR26172075	Plasma5	Genomic	Targeted-capture	Random	Paired
SRR26172076	Plasma4	Genomic	Targeted-capture	Random	Paired
SRR26172077	Plasma3	Genomic	Targeted-capture	Random	Paired
SRR26172078	Plasma2	Genomic	Targeted-capture	Random	Paired
SRR26172079	Plasma1	Genomic	Targeted-capture	Random	Paired
SRR26172080	Cap4_P2	Genomic	Targeted-capture	Random	Paired
SRR26172081	Cap3_T2	Genomic	Targeted-capture	Random	Paired
SRR26172082	Cap2_P1	Genomic	Targeted-capture	Random	Paired
SRR26172083	Cap1_T1	Genomic	Targeted-capture	Random	Paired

This table provides details of the second and third data sets. Among them, the types of Cap2_P1 and Cap4_P2 are Hepatitis B virus capture sequencing in liver tumor tissue, and the types of Cap1_T1 and Cap_T2 are Hepatitis B virus capture sequencing in liver paracancer tissue

## Data Availability

The first dataset was obtained through Illumina sequencing and was originally published by the Beijing Institute of Genomics (NCBI BioProject accession: PRJNA298941). The sequencing data of the second and third datasets were under the Bioproject ID PRJNA1011497 and they can be retrieved in the SRA (Sequence Read Archive, https://www.ncbi.nlm.nih.gov/sra) with run SRR26172074, SRR26172075, SRR26172076, SRR26172077, SRR26172078, SRR26172079, SRR26172080, SRR26172081, SRR26172082, and SRR26172083, respectively. The ViroISDC pipeline is available on https://github.com/QTazimi/ViroISDC.
